# Treatment of thoracic or lumbar burst fractures with Balloon Assisted Endplate Reduction using Tricalcium Phosphate cement: histological and radiological evaluation

**DOI:** 10.1186/s12891-017-1770-3

**Published:** 2017-10-10

**Authors:** Joep Kitzen, Martijn G. M. Schotanus, Herbert S. W. Plasschaert, Frans-Jan H. Hulsmans, Pieter B. J. Tilman

**Affiliations:** 1 Department of Orthopaedic Surgery, Zuyderland Medical Centre, Postbus 5500, 6130 MB Sittard-Geleen, the Netherlands; 2 Department of Pathology, Zuyderland Medical Centre, Sittard-Geleen, the Netherlands; 3 Department of Radiology, Zuyderland Medical Centre, Sittard-Geleen, the Netherlands

**Keywords:** Kyphoplasty, TCP, BAER, Biocompatibility, Recurrent kyphosis

## Abstract

**Background:**

Short-segment pedicle-screw instrumentation is frequently used to stabilize thoracolumbar burst fractures. A recognized disadvantage of this procedure is recurrent kyphosis from intervertebral disc creep into the fractured central endplate. Balloon Assisted Endplate Reduction (BAER) using Tricalcium Phosphate bone cement (TCP) enables elevation of the centrally depressed endplate. Our objective was to evaluate the bone-tissue response to TCP and to analyse whether BAER using TCP can prevent recurrent kyphosis after removal of the instrumentation.

**Methods:**

Fourteen patients with traumatic thoracolumbar burst fractures were operated with BAER using TCP in combination with short-segment instrumentation. Nine months after surgery, instrumentation was removed and transpedicular biopsies were taken for histological and histochemical analysis. Roentgenograms pre- and postoperatively and at latest follow-up after removal of the instrumentation were evaluated.

**Results:**

Average follow-up was 2.6 years. Analysis of the biopsies showed a variable degree of bone remodelling with incorporation of TCP into newly formed bone matrix. No extensive foreign body reactions, inflammation, granulomatous responses or tissue necrosis were observed. Wedge-angle, kyphosis-angle and both the anterior-posterior and central-posterior vertebral body height ratios improved significant postoperatively (*p* < 0.001). After removal of the instrumentation no significant differences in wedge-angle or height ratios were seen (*p* = 0.12). The kyphosis-angle increased four degrees (*p* = 0.01).

**Conclusion:**

TCP showed good histological osseointegration with no adverse events. TCP can therefore be safely used and could be beneficial in treatment of thoracolumbar burst fractures. BAER with TCP in combination with short-segment instrumentation might reduce recurrence of deformity even after removal of the instrumentation in comparison to short-segment instrumentation alone.

**Trial registration:**

This study is registered at the at the Dutch Trial Registry (NTR3498).

## Background

The best treatment option for thoracic- and lumbar burst fractures without neurological deficits is unclear. Several operative methods exist, but none has proven to be superior in terms of patient outcome. Treatment objectives after thoracic- and lumbar burst fractures are to stabilize the spine, prevent neurological deterioration, restore sagittal balance, retain as much segmental mobility as possible with the least operative tissue trauma and to mobilize the patient as quickly as possible.

Short-segment pedicle-screw instrumentation is widely used and can be done minimally invasive. However a recognized disadvantage of this procedure is early instrumentation failure [1, 2] and recurrent kyphosis [3]. Presumably because the posterior instrumentation alone does not provide sufficient support to allow the fractured vertebral body to heal without supplemental load-sharing through anterior column reconstruction. This anterior column insufficiency results form collapse of disc space and not from collapse of the fractured vertebral body [4, 5]. Öner et al. demonstrated that this collapse of disc space results from creep of the intervertebral disc into the fractured central endplate [6–8].

Posterior spinal fusion has been used to augment the stability of posterior implants. Several studies recommend posterior fusion, [9, 10] but others have claimed that it affords no benefit [11, 12]. A recent randomized trial of Jindal et al. [13] compared short-segment pedicle screw fixation with or without fusion of the affected segment. They showed a significant longer duration of surgery and higher peri-operative blood transfusion requirements in the fusion group. Whereas there were no clinical or radiological differences between the two groups.

Several studies evaluated the use of transpedicular vertebral body fracture reduction with Balloon Assisted Endplate Reduction (BAER) using Tricalcium Phosphate bone cement (TCP) and short-segment pedicle-screw instrumentation in thoracic- or lumbar burst fractures [14–17]. These studies demonstrated substantial reduction of the segmental kyphosis, presumably because BAER in combination with TCP cement prevents intervertebral disc creep by enabling elevation of the centrally depressed endplate. None of these studies reported on maintaining reduction after removal of the instrumentation. Removal of the instrumentation seems favourable in the lumbar spine since in non-fused segments without traumatic disc injury, segmental mobility presumably returns after the instrumentation is removed. Yurac et al. demonstrated residual mobility in these segments after the instrumentation was removed approximately 11 months after the initial posterior stabilisation for thoracolumbar burst fractures [18].

TCP cement rather than polymethylmethacrylate cement (PMMA) was used in the previously mentioned studies [14–17], because of its biocompatible properties shown in animal models [19, 20]. Biomechanically, TCP- and PMMA reinforced vertebrae have demonstrated nearly identical failure loads under compression [21, 22]. The use of the biocompatible TCP cement seems favourable. To our knowledge no studies on the biocompatibility of TCP cement for kyphoplasty in the human spine have been reported.

The main objectives of this retrospective cohort study were to evaluate the bone-tissue response of TCP cement in the human spine and to analyse whether BAER using TCP in combination with short-segment instrumentation for traumatic thoracic- or lumbar burst fractures can prevent recurrent kyphosis after removal of the instrumentation.

## Methods

The current study was approved by the Zuyderland medical ethics committee (13 N31) and registered at the Dutch Trial Registry (NTR3498). Informed consent was acquired in all patients. Medical records of patients who sustained an acute traumatic thoracolumbar burst fracture. (AO-type A.3 or B.1) between 2003 and 2010 were reviewed. The fractures were classified on CT-scan according to the AO-Magerl classification [23] and the load sharing classification (LSC) [1]. In 14 patients a BAER with TCP and short-segment pedicle-screw instrumentation was performed and consequently were included for analysis.

### Patients

The study group included eight female and six male patients with an average age of 41 years (range 23 to 61 years). The mean follow up was 29 months (range 9 to 100 months). All patients were operated between 0 to 21 days post injury (average 5 days). The mechanisms of injury were a fall from a height (*N* = 6), a traffic accident (*N* = 3) and domestic trauma (*N* = 5). The level of spinal involvement and type of fracture are listed in Table 1.

**Table 1:** Level of spinal involvement, fracture classification and type of procedure

Patient	Level of spinal involvement	Fracture classifaction^1^	Load sharing classification^3^	Type of procedure
1	L1 and L2	A3.1 and A3.2^2^	5 and 7^2^	Open procedure
2	L2 and L3	A3.2 and A1.2^2^	8 and 3^2^	Open procedure
3	L1	A3.1	5	Open procedure
4	L2	A3.2	7	Open procedure
5	L1	A3.3	9	MIS^4^
6	T12	A3.1	5	MIS^4^
7	L1	A3.1	5	MIS^4^
8	L4	A3.3	7	MIS^4^
9	L2	A3.2	7	MIS^4^
10	L2	A3.1	5	MIS^4^
11	L1	A3.1	7	MIS^4^
12	L3	A3.2	6	MIS^4^
13	Th11	A3.2	8	MIS^4^
14	L2	B1.2	7	MIS^4^

^1^AO-Magerl classification

^2^Respectively

^3^McCormack load sharing classification

^4^Minimally invasive surgery

Two patients had a spinal involvement of two levels. One patient sustained a burst fracture in both L1 and L2. The second patient sustained a burst fracture of L2 combined with a small compression fracture of L3 (AO-type A1.1). None of these patients had neurological deficits, pre-existing spinal deformity, spinal stenosis, osteoporosis (criteria WHO) or previous spinal surgery.

### Surgical procedure

All patients were operated on by the senior author (PT). Surgical procedures were performed with the patients under general anaesthesia with endotracheal intubation. Patients were placed in a prone position. Pedicle screws were placed into the non-fractured vertebrae one above and one below the fractured vertebrae. Consequently in the patients with 2 levels of spinal involvement, 3 instead of 2 segments were stabilised. Subsequently reduction of the kyphosis was achieved by inflation of the Kyphon® balloon-kyphoplasty in the fractured vertebrae (Medtronic, Minneapolis, Minnesota, USA). In the patient with a burst fracture in both L1 and L2, balloon-kyphoplasty was applied in both vertebrae. Stabilisation was achieved with an open procedure without fusion in the first 4 consecutive patients. Subsequently a percutaneous procedure was performed in the following 10 patients. The Monarch polyaxial spinal fixation device® and the Expedium Viper minimally invasive pedicle screw system® (Depuy, Warsaw, Indiana, USA) were used (Table 1). No distraction over the instrumentation was applied to achieve fracture reduction. For the cement augmentation, Calcibon® (Biomet, Warsaw, Indiana, USA) was used in the first 8 patients. In the subsequent 6 patients Kyphos® (Medtronic, Minneapolis, Minnesota, USA) was applied. The volume of injected TCP per vertebra ranged from 3 to 6 mL.

In 12 patients transpedicular biopsies from the bone-cement interface were taken during removal of the instrumentation using a Jamshidi needle. Two patients refused a biopsy. Instrumentation was removed on average 9 months (range 6 to 14 months) after the initial surgery. At that time-point consolidation of the fractured vertebra was expected based on radiological evaluation.

### Histological and histochemical analysis

Analysis of the biopsies was performed by a single pathologist (HP). The biopsies were fixated using 4% buffered formalin and subsequently decalcified with Ethylenediaminetetraacetic Acid. After paraffin embedding, histological slices were cut and stained with haematoxylin and eosin. Goldner, elastica von Gieson, Giemsa, periodic acid shift and/or reticulin stains were used to visualize the osteoid formation, the amount of fibrosis or to identify the cell population present in the biopsies. One additional sample consisting only of cement was processed in the same way as the biopsies for histological examination. The decalcified remnants of the cement were compared with the bone biopsies and used to identify the remnants of cement in the samples. The biopsies were scored for the presence of osteoid formation, woven bone, osteoclasts and osteoblasts using a score of one to three. One stands for a little amount or near absence of the features, two representing medium changes and three for a large amount or obvious features. The sum of these two results was made to determine the amount of bone remodeling (score of two to six). Finally, the amount of haematopoietic tissue present was measured (score of one to three).

### Radiological evaluation

**Fig. 1:**
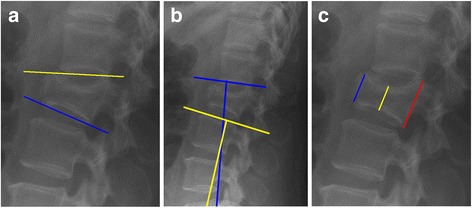
**a** *WA:* Angle between the superior- and inferior endplate of the fractured vertebra. **b** *SK:* Angle between the adjacent vertebral endplates of the fractured vertebra. **c** *APVBHr resp. CPVBHr:* Ratios between the anterior- resp. central vertebral height in relation to the posterior vertebral height

For study purposes standing anteroposterior and lateral roentgenograms pre- and approximately 9 months postoperatively and at latest follow-up after removal of the instrumentation were analysed (3 to 91 months after removal of the instrumentation). In eight out of the 12 patients with lumbar fractures, flexion-extension roentgenograms were available at latest follow-up. All roentgenograms were evaluated by two independent observers who were not involved in patient care (JK, FH). Mean values of their measurements were calculated. Interclass correlation coefficient was used to determine the conformity between the two observers. The wedge angle (WA, Fig. 1a), the segmental kyphosis angle (KA, Fig. 1b) and the ratios between both the anterior- and the posterior vertebral body height (APVBHr, Fig. 1c) and the central- and posterior vertebral body height (CPVBHr, Fig. 1c) were determined. Residual mobility was reported when both observers noted more than three degrees of range of motion between the flexion and extension roentgenograms in either the proximal or distal adjacent segment. Range of motion was defined as described by Tanz et al. [24] CT-scans were taken before and after the initial surgery to evaluate spinal canal encroachment, cement leakage and malposition of the instrumentation in all patients except for one.

### Patient reported outcome

After removal of the instrumentation at latest follow-up, back pain intensity was recorded on the basis of a Pain Visual Analog Scale (VAS, 10 point scale). Functional outcome after surgery was evaluated using the Short Form-36 survey (SF-36, domains physical function and bodily pain) and the Roland Morris Disability Questionnaire score (RDQ). Younger patients might show better compensation mechanisms after thoracolumbar fractures. A comparison between patients older than 40 years was made.

### Data analysis

Statistical analysis was performed with use of SPSS (Version 23.0). The non-parametric Wilcoxon Signed-Rank test was used for changes of each radiographic parameter. The level of significance was set at *p* < 0.05.

## Results

### Histological and histochemical analysis

**Fig. 2:**
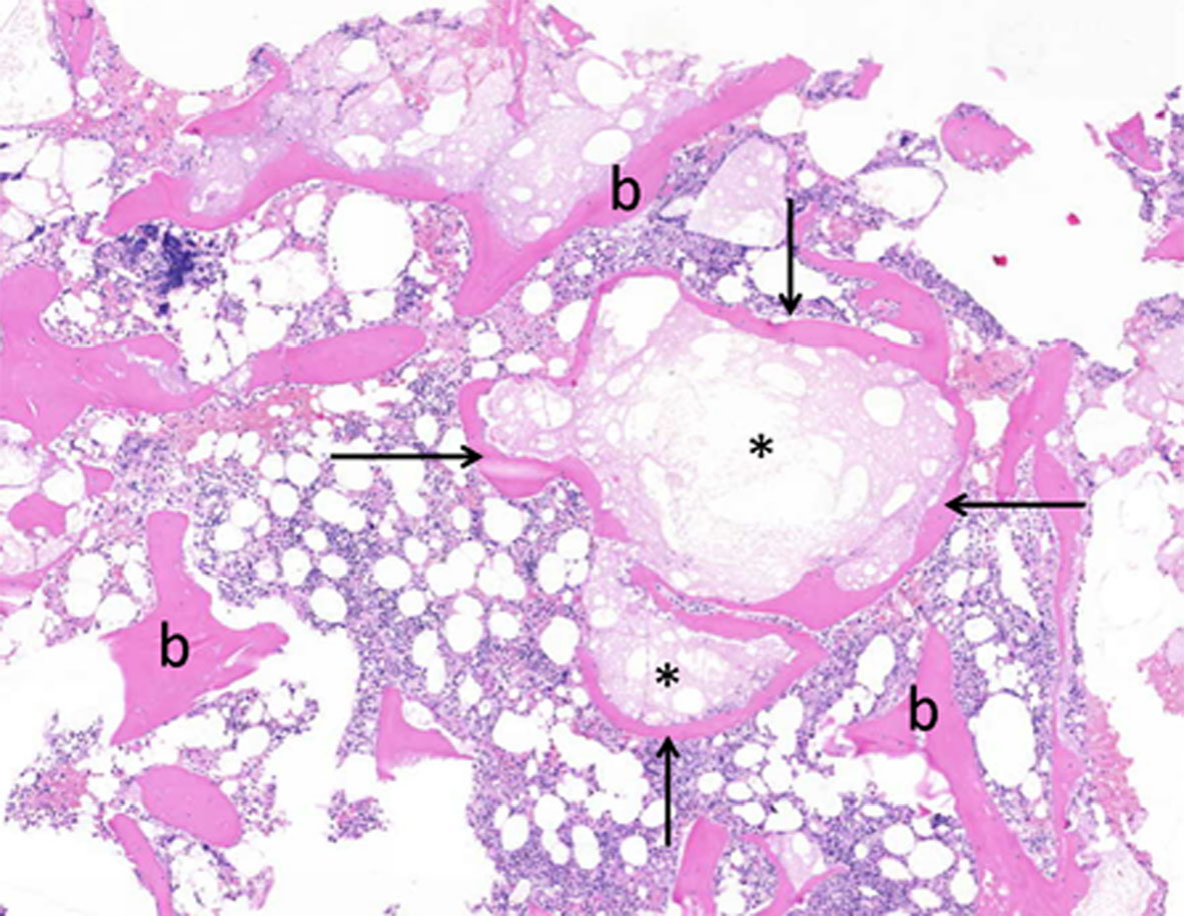
Island of bone (arrows) surrounding remnants of cement (*), compared to the usual bone trabeculae (b)

**Fig. 3:**
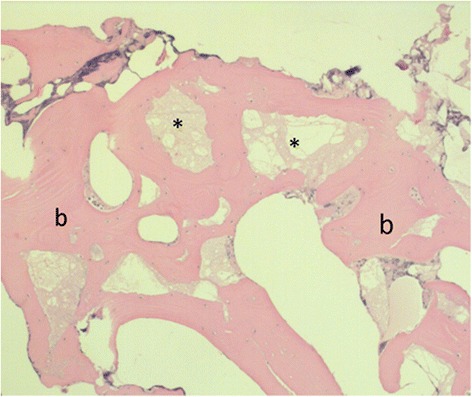
The result of bone remoddeling is shown in this picture, with remnants of the cement (*) and very broad bony trabeculae(b)

Twelve bone biopsies, taken 6 to 14 months after the initial surgery, were processed for histological examination. One sample was lost due to technical failure. In all of the 11 remaining biopsies, remnants of the cement were easily identified (Table 2). The presence of newly formed bone in the biopsies was striking (Figs. 2 and 3). This newly formed bone is deposited around cement particles with multiple osteoclasts and osteoblasts surrounding the particles. Thus producing islands and strands of bone, illustrating the osteoconductive properties of the TCP.

**Table 2:** Histological and histochemical analysis of the transpedicular biopsies from the bone-cement interface

Patient	Elapsed time (in months)^a^	Osteoid formation^b^	Osteoclasts & osteoblasts^b^	Combined^c^	Residual cement^b^	Haematopo-ietic tissue^b^
1	6	3	3	6	1	1
2	6	2	2	4	2	3
3	7	2	1	3	1	1
4	7	2	2	4	3	2
5	8	2	2	4	3	1
6	8	3	3	6	3	1
7	8	2	1	3	2	3
8	9	3	3	6	3	1
9	9	2	2	4	1	1
10	9	1	1	2	3	1
11	14	2	3	5	3	1

^a^Elapsed time between the initial surgery and the removal of the instrumentation

^b^Score of 1 to 3

^c^Score of 2 to 6

**Fig. 4:**
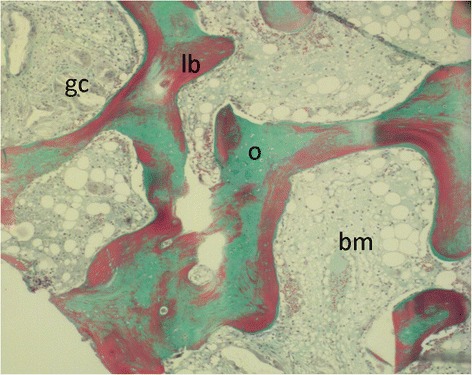
Goldner staining: Osteid (newly formed bone) colours green (o), mineralised bone stains red (b). The bone marrow in between is not stained (bm), although some giant cell reaction can be seen (gc). This is illustrated in more detail in fig. 5

As shown in Table 2, there is no clear correlation between the time of biopsy and the degree of bone remodelling. Extensive remodelling (score of 6) was found at 6 months and at 9 months (Fig. 4). On the other hand no remodelling (score of 2) was found in other biopsies taken at 7 or 9 months. No histological differences between the two types of cement (Calcibon and Kyphos) were observed.

**Fig. 5:**
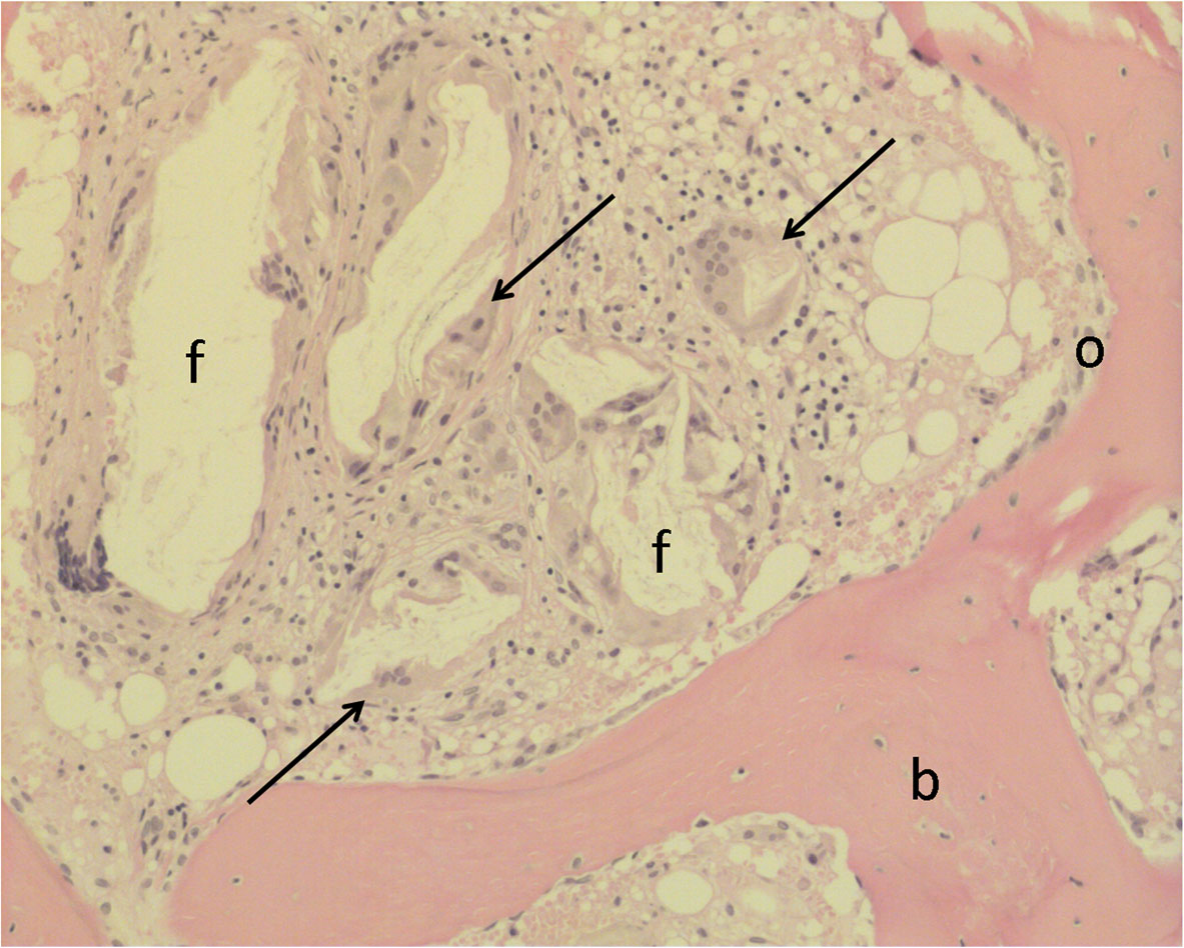
Focal giant cell reaction (arrow), the multinucleated giant cells form aggregates and engulf some foreign body material (f), presenting as empty space in the histology. Osteoblastic rimming (o) of a bone lamella (b) is seen next to it

The hematopoietic tissue situated in between the bony trabeculae and between the trabeculae and the remnants of the cement showed no striking aberrations (Table 2). No cellular discrepancies were identified when examining the megakaryopoiesis, myelopoiesis and erythropoiesis. One biopsy showed a marked but localized histiocytic giant cell reaction with ingestion of some debris in the multinucleated cells indicative for the resorption of TCP (Fig. 5). In all other biopsies extensive foreign body reactions, inflammation, granulomatous responses or tissue necrosis were absent.

### Radiological evaluation

The mean values for the reduction of the segmental kyphosis angle and restoration of the central endplates pre- and postoperatively and after removal of the instrumentation are displayed in Table 3. Interclass correlation coefficient for all angles and ratios measured between two observers (JK, FH) ranged between 0.83 and 0.95. Pre-operative wedge- and segmental kyphosis angle as well as both the APVBHr and CPVBHr improved significantly (*p* < 0.001) after the initial surgery. No signs of spinal canal encroachment, cement leakage or malposition of the instrumentation were seen on CT-scan. Convection was similar among patients with a percutaneous- or open posterolateral procedure.

**Table 3:** Mean values for the reduction of the segmental kyphosis angle and restoration of the central endplates pre- and postoperatively and after removal of the instrumentation

	Before surgery (*n* = 14)	Before removal instrumentation (*n* = 14)	*P*-value^1^	After removal instrumentation (*n* = 14)	*P*-value^2^
Wedge Angle (in degrees)	19 (8.8)	9 (6.2)	0,000	10 (6.8)	0.25
Segmental kyphosis angle (in degrees)	9 (6.8)	0 (7.8)	0,000	4 (7.9)	0.01
Central-posterior vertebral body height ratio	0.4 (0.2)	0.8 (0.1)	0,001	0.8 (0.1)	0.26
Anterior-posterior vertebral body height ratio	0.6 (0.2)	0.8 (0.1)	0,000	0.8 (0.1)	0.12

All values are shown as average (standard deviation)

^1^findings after the initial surgery compared with those before removal of the instrumentation

^2^findings after the initial surgery compared with those after removal of the instrumentation

After removal of the instrumentation the wedge angle as well as both height ratios were preserved. The segmental kyphosis angle increased significantly with a mean of four degrees (Table 3). However a significant reduction of five degrees (*p* = 0.01) was maintained in comparison to the preoperative kyphosis angle.

**Fig. 6:**
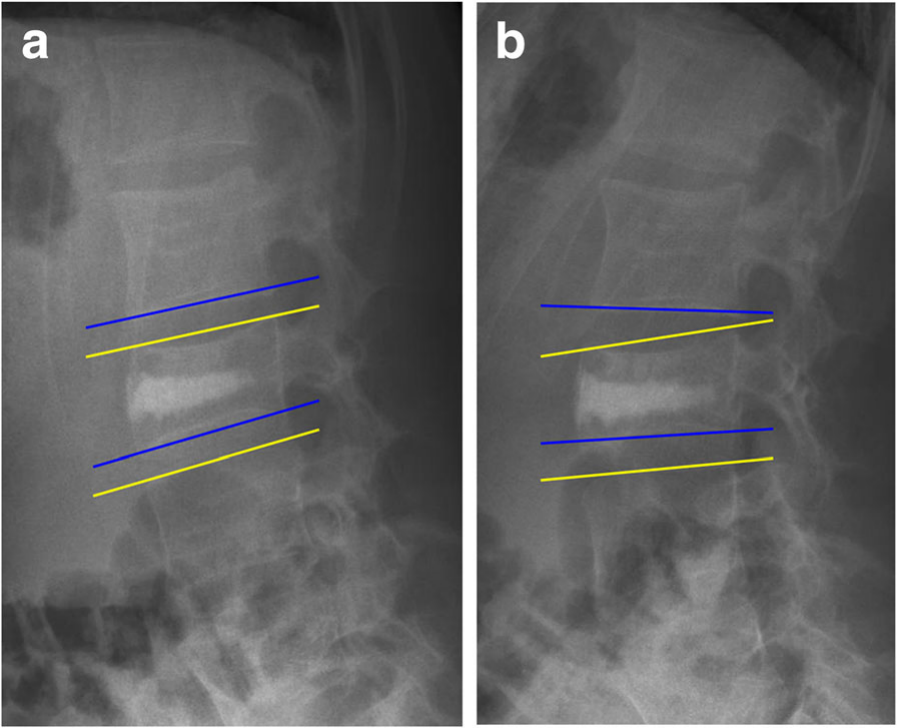
Flexion- (**a**) and extension (**b**) roentgenograms after removal of the instrumentation visualizing residual mobility

On roentgenograms at latest follow-up no spontaneous fusion was observed. However residual mobility (Fig. 6) was observed in six patients out of the eight patients in whom flexion-extension radiographs were obtained. This residual mobility was most pronounced in the distal adjacent segment, although as seen in Fig. 6 it was present at the proximal adjacent segment as well.

### Patient reported outcome

Average VAS pain scale after removal of the instrumentation was 2.8 (SD, 2.3). The SF-36 score and the RDQ score were respectively 72 (SD, 21) and 5.3 (SD, 5.9). When the patients older than 40 years (*n* = 7) in our group were compared to those younger (*n* = 7) no significant changes in VAS- (*p* = 0.33), RDQ- (*p* = 0.36) and SF-36 score (*p* = 0.44) were seen. With the exception of screw breakage in one patient, no other complications occurred (Table 1, patient 8). It must be noted that the patient with the screw breakage was a drug addict. In terms of both functional outcome and reduction of the segmental kyphosis this patient performed less.

## Discussion

In the current study the biocompatible properties of the TCP cement in the human spine were studied. In addition we evaluated if BAER using TCP cement in combination with short-segment instrumentation, could prevent recurrent kyphosis after removal of the instrumentation in patients with thoracic- or lumbar burst fractures.

Recurrence of deformity after short-segment instrumentation for thoracic- or lumbar burst fractures may result from intervertebral disc creep into the fractured central endplate [6–8]. A cadaver study by Verlaan et al. demonstrated that BAER with TCP cement significantly decreases the impression of the central endplate [25]. Indirect reduction of the fractured vertebral body by distraction using pedicle-screw instrumentation alone, leads to an insufficient reduction of the centrally depressed endplate [26]. Mermelstein et al. provided biomechanical evidence that in lumbar burst fractures, short-segment instrumentation in combination with transpedicular vertebral body reconstruction with TCP cement reduced pedicle screw-bending moments and increased the overall stiffness of the construct [27].

Several clinical studies evaluated the use of TCP for the reconstruction of osseous defects of the vertebral body, the distal part of the radius and the proximal part of the tibia and femur [16, 25, 28–30]. Verlaan et al. published a favourable histological bone response to TCP cement after vertebroplasty in goats [20]. The biopsies in our population showed comparable incorporation of the TCP cement into newly formed bone matrix, indicative for the biocompatible properties of the TCP cement. Extensive foreign body reaction, inflammation, granulomatous response or tissue necrosis were not seen. These findings are consistent with the study of Ooms et al. [19]. In this study calcium phosphate cement was injected as a paste into tibia cortical bone defects in goats. After histological evaluation they concluded that the material had good biocompatible properties. Furthermore TCP cement in contrast to PMMA is an endothermic reaction when it hardens. Exothermic reactions of the cement might induce necrosis and consequently degeneration of the discus [31].

Our study demonstrates a significant reduction of the segmental kyphosis after restoration of the central endplates when, in addition to the short-segment instrumentation, BAER with TCP was performed. These findings are consistent with other recently conducted studies [14–17]. A systematic review of Verlaan et al. demonstrated an average recurrent kyphosis (loss of reduction of the segmental kyphosis angle) of ten degrees in patients with thoracic or lumbar fractures treated with short-segment pedicle-screw instrumentation alone [3]. In the study of Jindal et al. [13] an average recurrent kyphosis of 5.5° was seen in the fusion group compared to 3.6° in the non-fusion group. The segmental kyphosis angles at latest follow-up were respectively 9.9° and 7.9°.

None of the previously mentioned studies report on the effects after removal of the instrumentation. A study by Öner et al. demonstrates a recurrent kyphosis of 10 degrees after removal of the instrumentation in patients with thoracolumbar fractures managed with posterior reduction and fusion [6]. In our series the restoration of the central impression of the endplates was maintained after removal of the instrumentation. A recurrent kyphosis of four degrees was observed after removal of the instrumentation. This loss of reduction occurred therefore probably in the discus space and was to our opinion not substantial, especially when compared to patients managed without BAER or with fusion of the affected segments.

Although in 6 out of 8 patients residual mobility was clearly observed, it is difficult to draw definitive conclusions since in only eight out of the 12 patients with lumbar fractures, flexion-extension roentgenograms were available. However this may speak in favor for removal of the material especially when the recurrent kyphosis afterwards is not substantial.

Based on the VAS scale, SF-36- and RDQ-score, functional outcome after removal of the instrumentation was good and comparable with the literature [14, 15]. Unfortunately no patient reported outcome measurements were obtained before removal of the instrumentation. It would have been interesting to be able to investigate if removal of the instrumentation is beneficial on functional outcome. With the exception of screw breakage and recurrence of deformity in one patient, there were no complications. This non-compliant patient had an LSC score of 7 and an AO-type A3.3 fracture, whereas most of the other patients sustained either an AO-type A3.1 or A3.2 fracture. Retrospectively a circumferential spondylodesis would have been a better treatment option when considering his compliance in relation to the severity of his fracture. McCormack et al. stated that spine fractures with low LSC scores (3 to 6 points) can be managed with short-segment posterior stabilization alone; whereas burst fractures with high LSC scores (7 to 9 nine points) require anterior stabilization to prevent early instrumentation failure [1]. In our series 9 out of the 14 patients had a LSC score of 7 or higher and only one non-compliant patient had early instrumentation failure. It seems that BAER gives sufficient support to the anterior column without supplemental anterior stabilisation. The main limitation of the current study is its retrospective nature and its small number of patients in combination with the heterogeneity of the population their follow-up.

## Conclusion

TCP cement showed histological osseointegration without adverse histological findings. TCP can therefore be safely used in treatment of thoracolumbar burst fractures. In addition this study indicates that BAER with TCP cement in combination with short-segment instrumentation might reduce the recurrence of deformity of the fractured vertebrae even after removal of the instrumentation when compared to short-segment instrumentation alone.

## Abbreviations

APVBHr: Anterior vertebral body height ratio
BAER: Balloon Assisted Endplate Reduction
CPVBHr: Central vertebral body height ratio
KA: Segmental kyphosis angle
LSC: Load sharing classification
MIS: Minimally invasive surgery
PMMA: Polymethylmethacrylate cement
RDQ: Roland Morris Disability Questionnaire score
SD: Standard deviation
SF-36: Short Form-36 survey
TCP: Tricalcium Phosphate bone cement
VAS: Pain Visual Analog Scale
WA: Wedge angle
